# Discrimination in the United States: Experiences of Latinos

**DOI:** 10.1111/1475-6773.13216

**Published:** 2019-10-30

**Authors:** Mary G. Findling, Sara N. Bleich, Logan S. Casey, Robert J. Blendon, John M. Benson, Justin M. Sayde, Carolyn Miller

**Affiliations:** ^1^ Department of Health Policy and Management Harvard T.H. Chan School of Public Health Boston Massachusetts; ^2^ Research, Evaluation, and Learning Unit Robert Wood Johnson Foundation Princeton New Jersey

**Keywords:** discrimination, Hispanic, Latinos, racial/ethnic differences in health and health care, racism, social determinants of health, survey research

## Abstract

**Objective:**

To examine experiences of racial/ethnic discrimination among Latinos in the United States, which broadly contribute to their poor health outcomes.

**Data Source and Study Design:**

Data come from a nationally representative, probability‐based telephone survey including 803 Latinos and a comparison group of 902 non‐Hispanic white US adults, conducted January—April 2017.

**Methods:**

We calculated the percent of Latinos reporting discrimination in several domains, including health care. We used logistic regression to compare the Latino‐white difference in odds of discrimination, and among Latinos only to examine variation by socioeconomic status and country of birth.

**Principal Findings:**

One in five Latinos (20 percent) reported experiencing discrimination in clinical encounters, while 17 percent avoided seeking health care for themselves or family members due to anticipated discrimination. A notable share of Latinos also reported experiencing discrimination with employment (33 percent applying for jobs; 32 percent obtaining equal pay/promotions), housing (31 percent), and police interactions (27 percent). In adjusted models, Latinos had significantly higher odds than whites for reporting discrimination in health care visits (OR: 3.18, 95% CI: 1.61, 6.26) and across several other domains. Latinos with college degrees had significantly higher odds of reporting discrimination in multiple domains than those without college degrees, with few differences between foreign‐born and US‐born Latinos.

**Conclusions:**

Latinos in the United States report experiencing widespread discrimination in health care and other areas of their lives, at significantly higher levels than whites. Being born in the United States and earning a college degree are not protective against discrimination, suggesting that further health and social policy efforts to eliminate discrimination are needed.

## 1 INTRODUCTION

In recent years, there has been a growing national debate about the seriousness of racial and ethnic discrimination in the United States.^1^ Despite this increased national attention, institutional discrimination against Latinos—the largest minority group in the United States^2^—has not been studied extensively with nationally representative data, nor across public policy domains. Because discrimination is associated with a wide range of adverse health outcomes and is therefore a prominent risk factor for Latinos' health,^3^, ^4^, ^5^, ^6^, ^7^ understanding the extent of discrimination against Latinos across different areas of their lives may partially explain observed variations in their health, particularly along relevant demographic factors affecting health.

Previous research suggests that nationality and socioeconomic status moderate the relationship between race/ethnicity and discrimination, but their effects depend on the racial or ethnic group of inquiry (eg, whites with higher education and income are less likely to report experiencing discrimination, while blacks and Asians with higher education and income are more likely to report discrimination).^8^, ^9^, ^10^, ^11^, ^12^, ^13^, ^14^ Additionally, 48 percent of Latino adults in the United States are foreign‐born,^15^ and previous studies suggest Latino groups have cultural differences related to ethnic identity, nativity, accent, and language that may be important correlates of discrimination.^13^, ^16^, ^17^

To date, the vast majority of existing studies on discrimination against Latinos have either relied on samples that cannot be generalized to the US Latino population (eg, small sample sizes, convenience samples, only Mexican Americans, only Latino immigrants),^8^, ^17^, ^18^ or have focused on everyday discrimination (unfair treatment)^13^ or discrimination within a single policy domain (such as health care)^11^, ^17^ and have not simultaneously examined the extent of discrimination Latinos face across multiple institutions or policy domains.

This study brings a public health perspective to the complexity and pervasiveness of discrimination in the United States today, alongside complementary articles in this issue of *Health Services Research*. It was conducted as part of a larger nationally representative survey fielded in 2017 in response to a growing national debate about discrimination in the United States today,^1^, ^19^ to understand experiences of discrimination against several different groups in America including blacks, Latinos, Asian Americans, Native Americans, women, and LGBTQ people. Specifically, the purpose of this study was threefold: (1) to document the prevalence of ethnic discrimination against Latinos adults across multiple institutional domains, including health care, education, employment, housing, political participation, police, and the criminal justice system, as well as interpersonal domains that affect health outcomes, including slurs, microaggressions, harassment, and violence; (2) to document disparities by comparing Latinos' experiences to whites; and (3) to examine the variation in Latinos' experiences of discrimination by socioeconomic status (income and education) and country of birth (US‐born vs foreign‐born).

## 2 METHODS

### 2.1 Study design and sample

Data were obtained from an original, nationally representative, probability‐based telephone (cell and landline) survey of US adults, conducted from January 26 to April 9, 2017. The survey was jointly designed by Harvard TH Chan School of Public Health, the Robert Wood Johnson Foundation, and National Public Radio. SSRS administered the survey. Because Harvard researchers were not directly involved in data collection and de‐identified datasets were used for analysis, the study was determined to be “not human subjects research” by the Harvard TH Chan School of Public Health Office of Human Research Administration.

The full sample included 3453 US adults aged 18 years and older, and this paper examines the subsample of 803 Latinos or Hispanics and 902 non‐Hispanic whites. Throughout the paper, we use shorthand descriptors of Latino or white. To identify Latinos and whites, screening questions regarding both racial and ethnic identity were asked at the beginning of the survey, and all questions about racial/ethnic identity were based on respondents' self‐identification. If respondents identified as Latino and another race, interviewers asked whether they identified more with being Hispanic/Latino (coded as Latino) or more with the other race (coded as the other race). In all follow‐up questions for Latino respondents, question wording used the term “Latino,” following language most commonly used. This method of screening also allowed interviewers to use the appropriate language in survey questions to describe or refer to the respondent's own identity. For example, this allowed questions to be read as “Did you experience [form of discrimination] because you are [‘Latino’]?” rather than “because of your race or ethnicity?” Interviewers were trained to identify Spanish‐speaking only respondents, and adults who preferred being interviewed in Spanish were interviewed by bilingual interviewers. Respondents were allowed to switch between Spanish and English according to their comfort level, and 38 percent of Latino surveys were conducted in Spanish (weighted percent).

The completion rate for this survey was 74 percent among respondents who answered initial demographic screening questions, with a 10 percent overall response rate, calculated based on the American Association for Public Opinion Research's (AAPOR) RR3 formula.^20^ Because data from this study were drawn from a probability sample and used the best available sampling and weighting practices in polling methods (eg, 68 percent of interviews were conducted by cell phone, and 32 percent were conducted via landline), they are expected to provide accurate results consistent with surveys with higher‐response rates^21^, ^22^ and are therefore reliably generalizable to the broader population, within a margin of error of ±4.5 percentage points at the 95% confidence interval. See Benson et al (2019)^23^ for a further description of the survey methodology.

### 2.2 Survey instrument

The poll asked about adults' experiences of discrimination. We conceptualized racial/ethnic discrimination as differential or unfair treatment of individuals based on self‐identified race or ethnicity, whether by individuals (based on beliefs, words, and behavior) or social institutions (based on laws, policies, institutions, and related behavior of individuals who work in or control these laws, policies, or institution).^4^, ^8^, ^24^ We analyzed 18 questions from the survey, covering six institutional and six interpersonal areas of discrimination (question wording in Appendix S1). Institutional areas included were health care; employment; education; housing; political participation; and police and courts. Interpersonal areas included were racial/ethnic slurs; microaggressions (ie, negative assumptions or insensitive or offensive comments about you); racial/ethnic fear; sexual harassment; being threatened or nonsexually harassed; and violence. We also examined two areas in which concerns about discrimination might prevent adults from taking needed action: seeking health services and protection from the police. We examined discrimination in domains previously demonstrated to be associated with health, as well as some that were not (eg, political participation), in order to capture a wide range of possible discriminatory experiences across adults’ lives. We also examined general beliefs about the existence of discrimination against one's own racial/ethnic group (Latinos or whites) in America today. Questions about experiences were only asked among a random half sample of respondents to maximize the number of questions while limiting respondent burden. Questions were only asked of relevant subgroups (eg, college questions only asked among adults who had ever applied to college). Questions on harassment, violence, and avoiding institutions for fear of discrimination were asked about yourself or family members because of the sensitive nature of the topic and prior literature demonstrating that vicarious experiences of stress (eg, through discrimination experienced by family members) can adversely affect individuals.^25^

### 2.3 Statistical analyses

After calculating descriptive statistics, we calculated the prevalence of all Latinos and whites who reported that they had ever experienced racial (white) or ethnic (Latino) discrimination in each of the domains. Using pairwise t tests of differences in proportions, we made uncontrolled comparisons of the percentage of Latino and white adults reporting discrimination across domains. For all analyses, statistical significance was determined at *P* < .05.

We then conducted logistic regression models to assess whether reporting discrimination remained significantly associated with race/ethnicity after controlling for the following variables that may be related to variation in experiences of discrimination^8^, ^9^, ^11^, ^13^, ^26^, ^27^: gender, age (18‐29, 30‐49, 50‐64, 65+), household income (<$25 000, $25 000‐<$50 000, $50 000‐<$75 000, $75 000+), education (less than college degree or college graduate), current health insurance status (used only for the health care question—uninsured, Medicaid insured, non‐Medicaid insured), neighborhood racial composition (measured as whether respondents live in a neighborhood that is predominantly their own race/ethnicity or not), metropolitan status (urban, suburban, rural), and region (US Census Bureau 4‐region division: Midwest, Northeast, South, West). Among Latino adults only, we estimated logistic regression models to examine variation in experiences of institutional discrimination by socioeconomic status (education and income) and country of birth, while controlling for gender, age, health insurance (for health care outcomes only), neighborhood racial/ethnic composition, and geographic measures. To test the sensitivity of our results to model specifications, we fit alternate models using different measures of discrimination, income, and education. We also tested models interacting age with income, but models showed insignificant results, most likely due to small sample size, and were ultimately dropped from analysis. In addition, among Latino adults only, we ran an ordinal logistic regression model to test characteristics associated with reporting overall institutional discrimination. Because questions were only asked among a randomized half sample of respondents, the scale of this model ran from 0 (no reported discrimination in any domains) to 7 (maximum possible reported discrimination in 7 different institutional domains).

To compensate for known biases in telephone surveys (eg, nonresponse bias) and variations in probability of selection within and across households, sample data were weighted by household size and composition, cell phone/landline use, and demographics (gender, age, education, race/ethnicity, and census region) to reflect the true population distribution of Latino and white adults in the country.^28^ Other techniques, including random‐digit dialing, replicate subsamples, and random selection of a respondent within a household, were used to ensure that the sample is representative. All analyses were conducted using STATA version 15.0 (StataCorp) and all tests accounted for the variance introduced by weighted data.

## 3 RESULTS

Weighted characteristics of Latinos and non‐Hispanic whites in this study sample are presented in Table 1. Latinos differed from whites on almost every demographic measure. Compared to whites, Latinos were younger, less likely to have a college degree (15 percent vs 34 percent, *P* < .01), and more likely to live in lower‐income households (less than $25 000 per year) (39 percent vs 23 percent, *P* < .01). Latinos were also more likely to lack health insurance than whites (22 percent vs 9 percent uninsured, *P* < .01), less likely to live in a neighborhood that was predominantly their own race/ethnicity (44 percent vs 67 percent, *P* < .01), and more likely to live in the Western United States (37 percent vs 18 percent, *P* < .01). About half of Latinos in this sample (49 percent) were born in the United States (including Puerto Rico).

**Table 1:** Characteristics of the study sample,by race/ethnicity

	Latinos (N = 803)^a^	Non‐Hispanic Whites (N = 902)^a^	*P*‐value for difference^b^
	Percent of respondents^c^		
Gender			
Male	50	48	.56
Female	50	52	.56
Age (y)			
18‐29	28	18	<.01*
30‐49	42	30	<.01*
50‐64	20	29	<.01*
65+	11	23	<.01*
Education			
No college degree^d^	85	66	<.01*
College degree or more	15	34	<.01*
Household income			
<$25 000	39	23	<.01*
$25 000‐<$50 000	24	22	.41
$50,000‐<$75,000	8	11	.07
$75 000+	17	35	<.01*
Don't know/refused	12	9	.10
Health insurance current status			
Uninsured	22	9	<.01*
Insured, Medicaid primary source	11	6	<.01*
Insured, non‐Medicaid primary source	66	84	<.01*
Living in a neighborhood that is predominantly own race/ethnicity^e^	44	67	<.01*
Area of residence			
Urban	22	17	.04*
Suburban	58	53	.10
Rural	12	25	<.01*
Don't know/refused	8	5	.25
US region of residence^f^			
Northeast	13	18	<.05*
Midwest	8	25	<.01*
South	34	35	.78
West	37	18	<.01*
Don't know/refused	7	4	.05
Country of birth			
Born in the United States^g^	49	—	—
Foreign‐born	51	—	—

Note

Latino and non‐Hispanic white adults ages 18+.

a The sample size shown reflects the total number of respondents in each category.

b *P*‐value for difference is based on *t* tests.

c Percent of US population estimated with survey weights to adjust for unequal probability of sampling, may not add up to 100% due to rounding.

d Includes those with some college experience (including business, technical, or vocational school after high school) but no college degree, as well as those with a high school degree or GED certificate or less.

e Question asked as: “People often describe some neighborhoods or areas as predominantly one group or another, such as a predominantly black or white neighborhood. Would you say that the area where you live is predominantly [Latino OR White], or not?”

f Regions defined by US Census Bureau 4‐region definition.

g Born in the United States includes those born in Puerto Rico. Question only asked of adults who identified as Latino/.

* Statistically significant difference between Latinos and whites at *P* < .05.

Table 2 shows unadjusted estimates of Latinos and whites reporting personal discrimination because of their race (whites) or ethnicity (Latinos) across institutional domains and interpersonal domains, as well as actions based on concerns about discrimination and perceptions of general discrimination against Latinos/whites in the US today. Overall, 78 percent of Latinos reported that “generally speaking, [they] believe discrimination against Latinos exists in America today,” compared to 55 percent of whites reporting they believe discrimination exists against whites in America today (*P* < .01). In the context of personally experiencing discrimination, Latinos were significantly more likely than whites to report experiencing discrimination in most institutional and interpersonal areas, including employment (applying for jobs: 33 percent vs 19 percent, *P* < .01, and obtaining equal pay or being considered for promotions: 32 percent vs 13 percent, *P* < .01); housing (trying to rent a room/apartment or buy a house: 31 percent vs 5 percent, *P* < .01), and hearing racial/ethnic slurs (37 percent vs 23 percent, *P* < .01) and microaggressions (33 percent vs 19 percent, *P* < .01). Latinos were more likely than whites to report experiencing discrimination in health care: both in going to a doctor or health clinic (20 percent vs 5 percent, *P* < .01) and avoiding the doctor or health care for themselves or family members due to concerns of discrimination or poor treatment (17 percent vs 3 percent, *P* < .01). Latinos were also more likely than whites to report discrimination with the police and courts: in police interactions (27 percent vs 10 percent, *P* < .01), that they or a family member had been unfairly stopped or treated by the police (27 percent vs 6 percent, *P* < .01) or unfairly treated by the courts (20 percent vs 7 percent, *P* < .01) because of their race/ethnicity, and that they had ever avoided calling the police because of concerns of discrimination (17 percent vs 2 percent, *P* < .01). Latinos were also more likely than whites to report discrimination in trying to vote or participate in politics (15 percent vs 4 percent, *P* < .01) and report others have acted afraid of them due to their race/ethnicity (15 percent vs 7 percent, *P* < .01).

**Table 2:** Differences between Latino and white adults in reporting discrimination because of race/ethnicity

	Subject of discrimination^a^	N	Latino percent^b^	White percent^b^	*P*‐value for difference^c^
*Belief in overall discrimination*					
General belief that discrimination against [your race/ethnicity] exists today in the United States^d^	All adults	1705	78	55	<.01*
*Personal experiences of institutional discrimination*					
Employment					
Applying for jobs^e^	You	791	33	19	<.01*
Being paid equally or considered for promotions^f^	You	800	32	13	<.01*
Education					
Applying to or while attending college^g^	You	615	19	11	.06
Health care					
Going to a doctor or health clinic	You	854	20	5	<.01*
Housing					
Trying to rent a room/apartment or buy a house^h^	You	664	31	5	<.01*
Political participation					
Trying to vote or participate in politics	You	851	15	4	<.01*
Police and Courts					
Interacting with police	You	851	27	10	<.01*
Unfairly stopped or treated by the police^i^	You or family member	851	27	6	<.01*
Unfairly treated by the courts^i^	You or family member	851	20	7	<.01*
*Personal experiences of interpersonal discrimination*					
Racial/ethnic slurs^j^	You	854	37	23	<.01*
Microaggressions^j^	You	854	33	19	<.01*
Racial/ethnic fear^j^	You	854	15	7	<.01*
Violence^i^	You or family member	851	20	13	.05
Threatened or nonsexually harassed^i^	You or family member	851	19	16	.38
Sexual harassment^i^	You or family member	851	11	9	.50
*Actions based on concerns about discrimination*					
Avoided doctor or health care because of concerns of discrimination/poor treatment	You or family member	854	17	3	<.01*
Avoided calling the police because of concerns of discrimination	You or family member	851	17	2	<.01*

Note

Latino and non‐Hispanic white adults ages 18+. Individual questions only asked among a randomized subsample of half of respondents within each racial/ethnic category. Don't know/refused responses included in the total for unadjusted estimates.

a Questions about you are personal experiences only; questions about you or family member ask if items have happened to you or a family member because you or they are [Latino OR White]. All adults asked about discrimination against [Latinos OR Whites] in America today.

b Unadjusted percent, calculated using survey weights.

c *P*‐value for difference between unadjusted estimates using t tests.

d Question asked as “Generally speaking, do you believe there is or is not discrimination against [Latinos OR Whites] in America today?”

e Jobs question only asked among respondents who have ever applied for a job.

f Equal pay question only asked among respondents who have ever been employed for pay.

g Colllege application/attendance was only asked among respondents who have ever applied for college or attended college for any amount of time.

h Housing question only asked among respondents who have ever tried to rent a room or apartment, or to apply for a mortgage or buy a home.

i Question wording: “Do you believe that you or someone in your family has [experienced/been _____] because you or they are [Latino OR White].”

j Question wording: “In your day‐to‐day life, have any of the following things ever happened to you, or not?” and respondent indicated they had experienced this *and* believed this happened because they are [Latino OR White]. Racial/ethnic slurs = someone referred to you or a group you belong to using a slur or other negative word; Microaggressions = someone made negative assumptions or insensitive or offensive comments about you; Racial/ethnic fear = people acted as if they were afraid of you.

* Statistically significant difference between Latinos and whites at *P* < .05.

**Figure 1:**
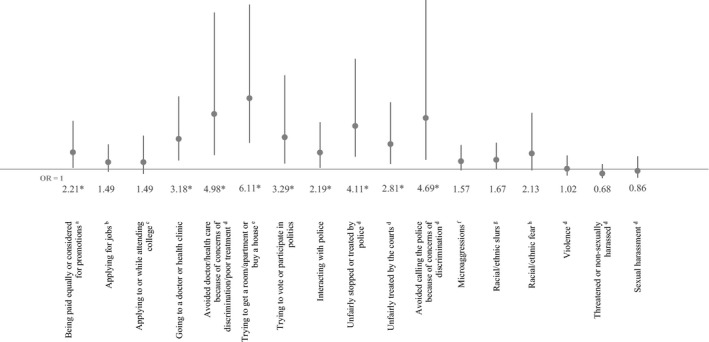
Adjusted odds of experiencing discrimination among Latinos compared to whites (reference group). OR, Odds Ratio, with 95% Confidence Interval bars. Nationally representative sample of Latino and non‐Hispanic White adults ages 18+. *Indicates statistical significance at *P* < .05. Don't know/refused responses coded as missing. Odds ratios report the odds that Latinos reported experiencing discrimination for each outcome (Whites were the reference group). These estimates control for gender, age (18‐29, 30‐49, 50‐64, 65+), education (<college vs college graduate or more), household income (<$25 k, $25 k‐<$50 k, $50 k‐<$75 k, $75 k+), living in a neighborhood that is predominantly one's own race/ethnicity, household location (urban, suburban, rural), region (Northeast, Midwest, South, West), and for health care outcomes only, health insurance status (uninsured, Medicaid insured, non‐Medicaid insured). ^a^Equal pay question only asked among respondents who have ever been employed for pay. ^b^Jobs question only asked among respondents who have ever applied for a job. ^c^College application/attendance was only asked among respondents who have ever applied for college or attended college for any amount of time. ^d^Includes discrimination against you or a family member because you are Latino/White. ^e^Housing question only asked among respondents who have ever tried to rent a room or apartment, or to apply for a mortgage or buy a home. ^f^Microaggressions indicate that someone made negative assumptions or insensitive or offensive comments about you because you are Latino/White. ^g^Racial/ethnic slurs indicate that someone referred to you or your racial group using a slur or other negative word because you are Latino/White. ^h^Racial/ethnic fear indicates that people acted as if they were afraid of you because you are Latino/White

After we controlled for potential sociodemographic confounders in logistic regression models (gender, age, education, household income, neighborhood racial composition, metropolitan status, region, and health insurance status where applicable), all Latino‐white disparities in reported institutional discrimination persisted except for job applications, while disparities in interpersonal discrimination were no longer statistically significant. Figure 1 shows the adjusted differences in the odds of Latinos personally experiencing discrimination compared to whites. The odds of Latinos reporting discrimination were more than four times the odds of whites when trying to rent a room or buy a house (OR [95% CI] 6.11 [2.89, 12.90]), avoiding health care due to discrimination concerns (4.98 [2.01, 12.32]), avoiding the police due to discrimination concerns (4.69 [1.67, 13.22]), and being unfairly treated by the police (4.11, [1.88, 8.98]). Latinos had at least three times the odds of whites of reporting discrimination when trying to vote or participate in politics (3.29 [1.39, 7.78]), and when going to a doctor or health clinic (3.18 [1.61, 6.26]) Latinos also had higher odds than whites of reporting discrimination in unfair treatment by the courts (2.81, 1.36, 5.83]), obtaining equal pay and promotions (2.21 [1.09, 4.48]), and police interactions (2.19 [1.09, 4.38]).

Among Latinos only, there were some differences in odds of reporting discrimination by socioeconomic status (education and income) and country of birth, as shown in Table 3. Latinos with a college degree had significantly higher odds than those without a college degree for reporting discrimination when applying for jobs (3.93 [1.80, 8.58]), with the police (police interactions—2.19 [1.02, 4.73]; being unfairly stopped/treated by police—3.33 [1.57, 7.05]), and in health care (going to the doctor—2.64 [1.13, 6.18]; avoiding the doctor due to discrimination concerns—2.90 [1.21, 6.98]). In addition, among Latinos who had ever applied for college or attended college for any amount of time, those with a college degree reported higher odds of experiencing discrimination in college applications and attendance (6.94 [2.30, 20.96]) compared to those who reported applying for college or attending college but did not have a college degree. Latinos living in the highest‐income households (at least $75 000/year) had lower odds of reporting discrimination compared to those living in the lowest‐income households (less than $25 000 per year) in applying for jobs (0.36 [0.13, 0.96]), obtaining housing (0.18 [0.05, 0.66]), police interactions (0.29 [0.10, 0.79]), and reporting discrimination in the ordinal logistic regression model (0.26 [0.14, 0.46). Foreign‐born Latinos had three times the odds of US‐born Latinos of reporting discrimination in obtaining equal pay and promotions (3.01 [1.43, 6.36]), but no differences in other domains.

**Table 3:** Odds of reporting personal experiences of ethnic discrimination across institutional domains among a national sample of Latino adults in the United States

	Employment		Education	Health care		Housing	Political participation	Police and courts				Overall institutional discrimination
	Applying for jobs^b^	Equal pay/promotions^c^	College application/attendance^d^	Doctor or health clinic visits	Avoided doctor due to discrimination concerns	Trying to rent or buy a house^e^	Trying to vote or participate in politics	Interacting with police	Unfairly stopped or treated by the police	Unfairly treated by the courts	Avoided calling the police due to discrimination concerns	Discrimination across 0‐7 domains^f^
N^a^	324	328	202	309	309	221	344	353	361	358	361	676
*OR (95% CI)*												
Gender												
Female	Ref	Ref	Ref	Ref	Ref	Ref	Ref	Ref	Ref	Ref	Ref	Ref
Male	1.53 (0.82, 2.86)	1.52 (0.80, 2.91)	0.87 (0.37, 2.05)	**0.27** * (0.12, 0.60)	**0.33** * (0.14, 0.76)	0.87 (0.35, 2.17)	2.16 (0.99, 4.71)	**2.25** * (1.21, 4.20)	1.46 (0.79, 2.69)	1.76 (0.89, 3.46)	2.10 (0.93, 4.74)	0.97 (0.66, 1.42)
Education												
<College	Ref	Ref	Ref	Ref	Ref	Ref	Ref	Ref	Ref	Ref	Ref	Ref
College+	**3.93** * (1.80, 8.58)	2.02 (0.89, 4.58)	**6.94** * (2.30, 20.96)	**2.64** * (1.13, 6.18)	**2.90** * (1.21, 6.98)	0.83 (0.33, 2.10)	1.39 (0.56, 3.47)	**2.19** * (1.02, 4.73)	**3.33** * (1.57, 7.05)	1.78 (0.77, 4.08)	1.88 (0.69, 5.12)	**2.31** * (1.48, 3.60)
Income												
$<25 k	Ref	Ref	Ref	Ref	Ref	Ref	Ref	Ref	Ref	Ref	Ref	Ref
$25 k‐<50 k	1.09 (0.51, 2.35)	1.43 (0.64, 3.23)	0.55 (0.14, 2.18)	0.86 (0.39, 1.92)	0.42 (0.17, 1.05)	0.64 (0.24, 1.70)	1.18 (0.45, 3.09)	0.63 (0.28, 1.40)	0.86 (0.39, 1.89)	0.94 (0.39, 2.31)	0.39 (0.16, 0.98)	0.83 (0.52, 1.32)
$50 k‐<75 k	1.01 (0.29, 3.50)	1.96 (0.53, 7.22)	0.32 (0.05, 2.09)	0.27 (0.06, 1.24)	0.08 (0.01, 0.52)	0.35 (0.08, 1.56)	0.88 (0.23, 3.45)	0.87 (0.26, 2.96)	1.92 (0.62, 5.95)	0.94 (0.21, 4.28)	0.26 (0.04, 1.77)	0.71 (0.31, 1.64)
$75 k+	**0.36** * (0.13, 0.96)	0.45 (0.16, 1.27)	0.46 (0.09, 2.28)	0.31 (0.09, 1.04)	0.05 (0.01, 0.18)	**0.18** * (0.05, 0.66)	0.37 (0.10, 1.40)	**0.29** * (0.10, 0.79)	0.41 (0.17, 1.01)	0.40 (0.13, 1.27)	0.12 (0.02, 0.54)	**0.26** * (0.14 0.46)
Country of birth												
US/Puerto Rico	Ref	Ref	Ref	Ref	Ref	Ref	Ref	Ref	Ref	Ref	Ref	Ref
Foreign‐born	1.72 (0.86, 3.46)	**3.01** * (1.43, 6.36)	0.35 (0.11, 1.11)	0.50 (0.20, 1.22)	0.58 (0.22, 1.53)	1.42 (0.58, 3.44)	1.59 (0.65, 3.91)	1.06 (0.50, 2.25)	0.52 (0.26, 1.04)	0.58 (0.25, 1.32)	0.94 (0.35, 2.56)	0.79 (0.50, 1.24)

Note

Nationally representative sample of Latino adults ages 18+.

Abbreviation: CI, confidence interval; OR, Odds ratio.

a Individual questions only asked among a randomized half sample of respondents. Logistic regression models also control for the following variables not shown: gender (male/female), age (18‐29, 30‐49, 50‐64, 65+), area of residence (urban, suburban, rural), whether you live in a neighborhood that is predominantly Latino (Yes/No), and US region of residence (South, Northeast, Midwest, West). Models for health care outcomes also adjust for insurance status (uninsured, Medicaid insured, non‐Medicaid insured). Don't know/refused responses coded as missing.

b Jobs question only asked among respondents who have ever applied for a job.

c Equal pay question only asked among respondents who have ever been employed for pay.

d College application/attendance was only asked among respondents who have ever applied for college or attended college for any amount of time.

e Housing question only asked among respondents who have ever tried to rent a room or apartment, or to apply for a mortgage or buy a home.

f Ordinal logistic regression model with experiencing discrimination in 0‐7 institutional domains as the outcome; individual questions only asked among a randomized half sample of respondents, so the maximum number of times a respondent could report experiencing discrimination in institutional questions was 7.

* Significant at *P* < .05 (shown in bold font).

Bold denotes statistical significance at *P* < .05.

Our results also showed age and gender differences in some areas. Younger Latino adults (aged 18‐29 years) had higher odds of reporting discrimination than older adults (aged 65+ years) in college applications/attendance, avoiding the doctor, political participation, treatment by the police/courts, and in the ordinal logistic regression model (data shown in Appendix S2). Latino men had lower odds than women of reporting discrimination in health care, but higher odds of reporting discrimination in police interactions.

There were no differences in experiences of discrimination by whether Latinos lived in predominantly Latino neighborhoods, area of residence, or region of the United States, except higher odds of reporting avoiding the police due to anticipated discrimination among Latinos living in the West compared to the South. In the ordinal logistic regression model, having a college degree was associated with reporting more types of discrimination (2.31 [1.48, 3.60]). The relationship between education and discrimination was reversed among whites (data not shown). Full model results are shown in Appendix S2.

## 4 DISCUSSION

Four key findings emerged from this national survey of Latino adults. First, we found widespread reported discrimination against Latinos. A majority of Latinos perceived general discrimination against Latinos in America today, while one in five reported experiencing discrimination in their clinical encounters.

Second, we found major differences in discrimination experienced between Latinos and whites. Regardless of socioeconomic status, Latinos reported experiencing discrimination at significantly higher levels than whites in health care and several other social institutions, including in clinical encounters and avoiding seeking health care due to anticipated discrimination.

Third, our results also showed that education was not protective against discrimination for Latinos in any policy domain, and instead was associated with *higher* levels of discrimination against Latinos in their jobs, police interactions, health care, and college. This relationship was reversed among whites, where higher socioeconomic status was associated with reporting less discrimination. These results are consistent with prior literature showing that high‐SES minorities may experience greater discrimination than their lower‐SES counterparts.^8^, ^10^, ^11^, ^12^ However, our results do not explain why this might occur. It is unclear whether this relationship is driven by unequal exposures (eg, high‐SES minorities having greater contact with whites than low‐SES minorities) or differential reporting (eg, high‐SES minorities being more likely to recognize and/or self‐report unequal treatment than low‐SES minorities).^14^, ^29^, ^30^ Future research should seek to explore the reasons for these findings among Latinos.

Fourth, we found few differences in discrimination between foreign‐born Latinos and US‐born Latinos after controlling for other major sociodemographic characteristics, suggesting that Latinos face discrimination, regardless of their nationality and immigration status.

While it is beyond the scope of these results to recommend specific approaches to ending discrimination, these findings update prior studies showing that Latinos continue to face widespread barriers to equal treatment across public institutions and interpersonally.^13^, ^18^ Our results add to the literature by showing that Latinos widely believe discrimination against their ethnic group occurs in the United States today, they continue to report experiencing high levels of discrimination personally, and more than one in six have avoided seeking health care and calling the police to avoid experiencing discrimination or unfair treatment.

Discrimination carries major health consequences for Latinos in the United States. Other research has found that prolonged or repeated discrimination causes major health problems over time, due to progressive wear and tear on the body's systems owing to repeated adaption to stressors (known as allostatic load and overload).^31^, ^32^ We expect that on average, discrimination will have a greater effect on the health of high‐socioeconomic status and upwardly mobile Latinos, who report experiencing greater discrimination than Latinos with lower‐socioeconomic status and those who are not upwardly mobile.^8^ Discrimination also carries major consequences for Latinos' opportunities for fair treatment in education, occupations, wages, medical care, and public safety.

Taken together, this literature suggests that equalizing access to physical, economic, and social resources are not enough to eliminate health disparities between Latinos and whites, and that further action to eliminate discrimination at the population level is needed.^5^ While there are major policy opportunities to address the discrimination documented in this study,^33^, ^34^, ^35^, ^36^ most interventions have not been rigorously evaluated for their effects on reducing racial/ethnic health disparities.

These results identify important future areas of research. For example, many of the observed differences in discrimination between Latinos and whites were not attributable to individual‐level demographic characteristics, suggesting that other factors at the household, neighborhood, community, state, or national levels may be important to consider (eg, family situation, occupation, county‐level poverty rate, political environment). Future discrimination studies should aim to continue using nationally representative samples and larger sample sizes where possible. Future work should also aim to include Latinos' racial, cultural, and geographic heritage, as there is considerably heterogeneity in the background and experiences of different Latino groups (eg, Mexican, Puerto Rican, Cuban).^37^ Future work should also identify, implement, and rigorously evaluate policy interventions to reduce discrimination against Latinos, as well as include eliminating discrimination as a clear aim in policies seeking to improve Latinos’ social, physical, and economic conditions.

### 4.1 Limitations

Our results should be interpreted considering several limitations. Interviews were conducted in both English and Spanish to overcome some communication challenges. Nonetheless, some respondents may have been excluded from the study due to language barriers, respondents may have interpreted questions differently based on varying backgrounds and expectations, and they may have reported experiences of cultural or communication problems as discrimination. Despite this, previous research has shown that discrimination affects health through multiple pathways and that perceived, self‐reported discrimination is associated with worse health outcomes.^4^, ^6^, ^8^ We did not ask about the timing or severity of experiences of discrimination. We also did not examine discrimination experienced as a result of respondents' other social identities (eg, LGBTQ‐based or gender‐based discrimination), or racial/ethnic identities they had in addition to the one they chose as their primary identity, which may compound experiences of discrimination among some adults. Our low response rate is a notable limitation, though evidence suggests that low response rates do not bias results if the survey sample is representative of the study population.^21^, ^22^ Recent research has shown that such surveys, when based on probability samples and weighted using US Census parameters, yield accurate estimates in most cases when compared with both objective measures and higher‐response surveys.^21^, ^22^, ^38^, ^39^ For instance, a recent study showed that across fourteen different demographic and personal characteristics, the average difference between government estimates from high‐response rate surveys and a Pew Research Center poll with a response rate similar to this poll was 3 percentage points.^21^ However, it is still possible that some selection bias may remain that is related to the experiences being measured. In addition, large confidence intervals in some logistic regression models (eg, discrimination in college applications/attendance) should be cautiously interpreted, as they may indicate low precision in some estimates. We also did not distinguish between the experiences of different Latino heritage groups (eg, Mexicans vs Cubans).

Despite these limitations, this study design allowed us to closely examine reported experiences of ethnic discrimination among Latinos. Our results highlight the extent of discrimination currently experienced by Latinos across public policy areas. Latinos face significant barriers to equal treatment across public institutions and policies, which carries major consequences for their health and well‐being.

## 5 CONCLUSIONS

A sizeable share of Latinos currently report experiencing discrimination in health care and many other areas of their lives at significantly higher levels than whites, which other research shows carries severe health consequences. Being born in the United States and earning a college degree are not protective against discrimination among Latinos. These results underscore a continuing need for deliberate health and social policy changes to attenuate and eliminate systemic discrimination against Latinos in the United States, beyond improving their social, physical, and economic conditions.
