# Supplementary material for: Discrimination in the United States: Experiences of Latinos
Source: Health Serv Res. 2019 Oct 30;54(Suppl 2):1409–18. doi: 10.1111/1475-6773.13216 (PMC6864375; doi:10.1111/1475-6773.13216)
Supplement: Supplementary file 2 [file HESR-54-1409-s002.docx]

**Appendix S1. Survey Questions**

**Screening questions**

1. Have you ever applied for a job?
2. (Half sample): Have you ever been employed for pay?
3. (Half sample): Have you ever applied for college or attended college for any amount of time?
4. (Half sample): Have you ever tried to rent a room or apartment, or to apply for a mortgage or buy a home?

**General perceptions of discrimination**

1. Generally speaking, do you believe there is or is not discrimination against [Latinos OR Whites] in America today? Yes / No / (Volunteered response) Don’t know/Refused

**Institutional Discrimination**

1. (Ask if respondent has ever applied for a job): What about you? Do you believe you have ever personally experienced discrimination because you are [Latino OR White] **when applying for jobs?** Yes / No / (Volunteered response) Don’t know/Refused
2. (Ask if respondent has ever been employed): What about you? Do you believe you have ever personally experienced discrimination because you are [Latino OR White] **when it comes to being paid equally or considered for promotions**? Yes / No / (Volunteered response) Don’t know/Refused
3. What about you? Do you believe you have ever personally experienced discrimination because you are [Latino OR White] **when interacting with police**? Yes / No / (Volunteered response) Don’t know/Refused
4. What about you? Do you believe you have ever personally experienced discrimination because you are [Latino OR White] **when trying to vote or participate in politics**? Yes / No / (Volunteered response) Don’t know/Refused
5. What about you? Do you believe you have ever personally experienced discrimination because you are [Latino OR White] **when going to a doctor or health clinic**? Yes / No / (Volunteered response) Don’t know/Refused
6. (Ask if respondent has ever applied to or attended college): What about you? Do you believe you have ever personally experienced discrimination because you are [Latino OR White] **when applying to college or while at college**? Yes / No / (Volunteered response) Don’t know/Refused
7. (Ask if respondent has ever tried to rent/buy a place to live): What about you? Do you believe you have ever personally experienced discrimination because you are [Latino OR White] **when trying to rent a room or apartment or buy a house**? Yes / No / (Volunteered response) Don’t know/Refused

**Interpersonal Discrimination (Against You Only)**

(Rotate items B and C, always ask A last): In your day-to-day life, have any of the following things ever happened to you, or not?

How about (INSERT)?

(IF RESPONDENT ASKS WHAT ‘GROUP’ MEANS, PLEASE SAY: Such as your race, ethnicity, gender (or your sexual orientation or identity).)

Yes, has happened / No, has not happened / (Volunteered response) Don’t know/Refused

9. Someone referred to you or a group you belong to using a slur or other negative word

10. Someone made negative assumptions or insensitive or offensive comments about you

11. People acted as if they were afraid of you

(Q9-Q11) (If Yes to previous question): Do you believe this happened to you because of your race or ethnicity, your gender, (your sexual orientation or gender identity,) or was it for some other reason? You can select multiple answers.

Race or ethnicity, Gender, Sexual orientation, Gender identity, Or some other reason (SPECIFY)

**Interpersonal Discrimination (Against You or Family)**

(Scramble items A-E; always ask B right after A)

Do you believe that you or someone in your family has (INSERT ITEM) because you or they are [Latino OR White]? How about (INSERT ITEM)?

Yes / No / (Volunteered response) Don’t know/Refused

12. Experienced sexual harassment

13. Been threatened or non-sexually harassed

14. Been unfairly stopped or treated by the police

15. Been unfairly treated by the courts

16. Experienced violence

**Avoiding health care**

17. Have you ever avoided going to a doctor or seeking health care for you or others in your family out of concern that you would be discriminated against or treated poorly because you or they are [Latino OR White]? Yes / No / (Volunteered response) Don’t know/Refused

**Avoiding police/law enforcement**

18. Have you ever avoided calling the police or other authority figures, even when in need, out of concern that you or others in your family would be discriminated against because you or they are [Latino OR White]? Yes / No / (Volunteered response) Don’t know/Refused

**Appendix S2*.* Full model showing the odds of reporting personal experiences of ethnic discrimination across institutional domains of discrimination among a national sample of Latino adults in the U.S.**

|  | **Employment** | | **Education** | **Health Care** | | **Housing** | **Political Partici-pation** | **Police and Courts** | | | | **Overall Institu-tional Discrim-ination** |
| --- | --- | --- | --- | --- | --- | --- | --- | --- | --- | --- | --- | --- |
|  | **Applying for jobs ^b^** | **Equal pay/ promotions ^c^** | **College application/ attendance ^d^** | **Doctor or health clinic visits** | **Avoided doctor due to discrimination concerns** | **Trying to rent or buy a house ^h^** | **Trying to vote or participate in politics** | **Interacting with Police** | **Unfairly stopped or treated by the police** | **Unfairly treated by the courts** | **Avoided calling the police due to discrimination concerns** | **Discrimination Across 0-7 Domains ^f^** |
| N ^a^ | 324 | 328 | 202 | 309 | 309 | 221 | 344 | 353 | 361 | 358 | 361 | 676 |
| OR (95% CI) | | | | | | | | | | |  |  |
| **Gender** | |  |  |  |  |  |  |  |  |  |  |  |
| Female | Ref | Ref | Ref | Ref | Ref | Ref | Ref | Ref | Ref | Ref | Ref | Ref |
| Male | 1.53  (0.82, 2.86) | 1.52  (0.80, 2.91) | 0.87  (0.37, 2.05) | **0.27***  (0.12, 0.60) | **0.33***  (0.14, 0.76) | 0.87  (0.35, 2.17) | 2.16  (0.99, 4.71) | **2.25***  (1.21, 4.20) | 1.46  (0.79, 2.69) | 1.76  (0.89, 3.46) | 2.10  (0.93, 4.74) | 0.97  (0.66, 1.42) |
| **Education** |  |  |  |  |  |  |  |  |  |  |  |  |
| <College | Ref | Ref | Ref | Ref | Ref | Ref | Ref | Ref | Ref | Ref | Ref | Ref |
| College+ | **3.93***  (1.80, 8.58) | 2.02  (0.89, 4.58) | **6.94***  (2.30, 20.96) | **2.64***  (1.13, 6.18) | **2.90***  (1.21, 6.98) | 0.83  (0.33, 2.10) | 1.39  (0.56, 3.47) | **2.19***  (1.02, 4.73) | **3.33***  (1.57, 7.05) | 1.78  (0.77, 4.08) | 1.88  (0.69, 5.12) | **2.31***  (1.48, 3.60) |
| **Income** |  |  |  |  |  |  |  |  |  |  |  |  |
| $<25k | Ref | Ref | Ref | Ref | Ref | Ref | Ref | Ref | Ref | Ref | Ref | Ref |
| $25k-<50k | 1.09  (0.51, 2.35) | 1.43  (0.64, 3.23) | 0.55  (0.14, 2.18) | 0.86  (0.39, 1.92) | 0.42  (0.17, 1.05) | 0.64  (0.24, 1.70) | 1.18  (0.45, 3.09) | 0.63  (0.28, 1.40) | 0.86  (0.39, 1.89) | 0.94  (0.39, 2.31) | 0.39  (0.16, 0.98) | 0.83  (0.52, 1.32) |
| $50k-<75k | 1.01  (0.29, 3.50) | 1.96  (0.53, 7.22) | 0.32  (0.05, 2.09) | 0.27  (0.06, 1.24) | 0.08  (0.01, 0.52) | 0.35  (0.08, 1.56) | 0.88  (0.23, 3.45) | 0.87  (0.26, 2.96) | 1.92  (0.62, 5.95) | 0.94  (0.21, 4.28) | 0.26  (0.04, 1.77) | 0.71  (0.31, 1.64) |
| $75k+ | **0.36***  (0.13, 0.96) | 0.45  (0.16, 1.27) | 0.46  (0.09, 2.28) | **0.31***  (0.09, 1.04) | 0.05  (0.01, 0.18) | **0.18***  (0.05, 0.66) | 0.37  (0.10, 1.40) | **0.29***  (0.10, 0.79) | 0.41  (0.17, 1.01) | 0.40  (0.13, 1.27) | 0.12  (0.02, 0.54) | **0.26***  (0.14 0.46) |
| **Country of birth** | |  |  |  |  |  |  |  |  |  |  |  |
| US/  Puerto Rico | Ref | Ref | Ref | Ref | Ref | Ref | Ref | Ref | Ref | Ref | Ref | Ref |
| Foreign Born | 1.72  (0.86, 3.46) | **3.01***  (1.43, 6.36) | 0.35  (0.11, 1.11) | 0.50  (0.20, 1.22) | 0.58  (0.22, 1.53) | 1.42  (0.58, 3.44) | 1.59  (0.65, 3.91) | 1.06  (0.50, 2.25) | 0.52  (0.26, 1.04) | 0.58  (0.25, 1.32) | 0.94  (0.35, 2.56) | 0.79  (0.50, 1.24) |
| **Covariates** |  |  |  |  |  |  |  |  |  |  |  |  |
| **Living in a Predominantly Latino Neighborhood** | | | |  |  |  |  |  |  |  |  |  |
| No | Ref | Ref | Ref | Ref | Ref | Ref | Ref | Ref | Ref | Ref | Ref | Ref |
| Yes | 1.89  (0.94, 3.76) | 1.89  (0.90, 3.96) | 0.43  (0.14, 1.31) | 1.54  (0.72, 3.28) | 1.09  (0.46, 2.60) | 0.43  (0.18, 1.03) | 1.21  (0.50, 2.96) | 0.89  (0.43, 1.87) | 0.84  (0.40, 1.74) | 0.70  (0.33, 1.51) | 0.57  (0.22, 1.45) | 1.10  (0.72, 1.67) |
| **Age** |  |  |  |  |  |  |  |  |  |  |  |  |
| 18-29 y | Ref | Ref | Ref | Ref | Ref | Ref | Ref | Ref | Ref | Ref | Ref | Ref |
| 30-49 y | 1.22  (0.52, 2.89) | **2.99***  (1.14, 7.83) | **0.27***  (0.09, 0.84) | 2.45  (0.83, 7.28) | 1.08  (0.34, 3.43) | 1.08  (0.32, 3.67) | 0.86  (0.33, 2.30) | 1.63  (0.69, 3.88) | 0.69  (0.32, 1.48) | 1.30  (0.49, 3.46) | 1.19  (0.36, 3.96) | 1.38  (0.79, 2.43) |
| 50-64 y | 1.14  (0.41, 3.18) | 1.94  (0.63, 5.99) | 0.77  (0.23, 2.61) | 1.53  (0.47, 4.97) | 1.68  (0.55, 5.12) | 0.61  (0.17, 2.12) | 0.42  (0.13, 1.43) | 1.02  (0.37, 2.85) | 0.52  (0.18, 1.53) | 0.73  (0.25, 2.09) | 1.17  (0.30, 4.46) | 0.90  (0.49, 1.65) |
| 65+ y | 0.59  (0.18, 1.89) | 0.63  (0.19, 2.03) | **0.05***  (0.004, 0.56) | 1.37  (0.33, 5.60) | **0.20***  (0.04, 0.98) | 0.39  (0.09, 1.60) | **0.09***  (0.02, 0.52) | **0.26***  (0.07, 0.95) | **0.18***  (0.06, 0.58) | **0.19***  (0.05, 0.68) | **0.11***  (0.02, 0.62) | **0.35***  (0.18, 0.68) |
| **Health Insurance** | |  |  |  |  |  |  |  |  |  |  |  |
| Uninsured | -- | -- | -- | Ref | Ref | -- | -- | -- | -- | -- | -- | -- |
| Medicaid | -- | -- | -- | 0.80  (0.17, 3.68) | 0.34  (0.05, 2.21) | -- | -- | -- | -- | -- | -- | -- |
| Non-Medicaid | -- | -- | -- | **0.36***  (0.14, 0.90) | 0.49  (0.18, 1.37) | -- | -- | -- | -- | -- | -- | -- |
| **Area of residence** | |  |  |  |  |  |  |  |  |  |  |  |
| Urban | Ref | Ref | Ref | Ref | Ref | Ref | Ref | Ref | Ref | Ref | Ref | Ref |
| Suburban | 1.66  (0.76, 3.61) | 1.35  (0.63, 2.86) | 0.79  (0.28, 2.23) | 0.42  (0.17, 1.03) | 0.32  (0.11, 0.93) | 0.62  (0.24, 1.58) | 1.26  (0.48, 3.33) | 1.45  (0.67, 3.15) | 1.07  (0.50, 2.30) | 0.81  (0.36, 1.80) | 0.46  (0.16, 1.31) | 0.88  (0.57, 1.37) |
| Rural | 1.29  (0.42, 3.93) | 0.87  (0.22, 3.48) | 1.78  (0.41, 7.73) | 0.57  (0.15, 2.23) | 0.13  (0.02, 0.86) | 0.39  (0.09, 1.66) | 2.52  (0.73, 8.61) | 1.77  (0.58, 5.39) | 1.77  (0.65, 4.80) | 0.93  (0.27, 3.24) | 0.15  (0.04, 0.54) | 1.15  (0.57, 2.33) |
| **Region of the Country** | |  |  |  |  |  |  |  |  |  |  |  |
| South | Ref | -- | -- | -- | Ref | -- | -- | -- | Ref | Ref | Ref | Ref |
| Northeast | 0.86  (0.33, 2.25) | 2.42  (0.95, 6.18) | 1.49  (0.46, 4.75) | 1.15  (0.41, 3.20) | 0.97  (0.30, 3.14) | 0.97  (0.28, 3.31) | 0.90  (0.26, 3.10) | 1.38  (0.52, 3.62) | 0.73  (0.28, 1.89) | 1.38  (0.50, 3.81) | 0.45  (0.11, 1.90) | 0.79  (0.46, 1.36) |
| Midwest | 1.26  (0.41, 3.85) | 0.44  (0.11, 1.75) | 0.51  (0.09, 2.84) | 0.74  (0.19, 2.80) | 1.55  (0.31, 7.78) | 0.85  (0.16, 4.37) | 1.23  (0.34, 4.49) | 1.93  (0.62, 6.05) | 0.79  (0.27, 2.31) | 0.80  (0.22, 2.93) | 3.33  (0.81, 13.66) | 0.95  (0.46, 1.96) |
| West | 0.70  (0.33, 1.47) | 1.07  (0.49, 2.34) | 2.33  (0.80, 6.78) | 2.19  (0.93, 5.18) | 1.34  (0.49, 3.63) | 1.34  (0.53, 3.39) | 0.83  (0.33, 2.09) | 1.29  (0.60, 2.80) | 1.04  (0.48, 2.24) | 2.06  (0.89, 4.74) | **4.54***  (1.41, 14.66) | 1.44  (0.90, 2.31) |

Table notes: *Significant at p<0.05. Nationally representative sample of Latino adults ages 18+. OR=Odds Ratio, CI=Confidence Interval. ^a^ Individual questions only asked among a randomized half sample of respondents. Don’t know/refused responses coded as missing. ^b^ Jobs question only asked among respondents who have ever applied for a job. ^c^ Equal pay question only asked among respondents who have ever been employed for pay. ^d^ College application/attendance was only asked among respondents who have ever applied for college or attended college for any amount of time. ^e^ Housing question only asked among respondents who have ever tried to rent a room or apartment, or to apply for a mortgage or buy a home.  ^f^ Ordinal logistic regression model with experiencing discrimination in 0-7 institutional domains as the outcome; individual questions only asked among a randomized half sample of respondents, so the maximum number of times a respondent could report experiencing discrimination in institutional questions was 7.
